# Audit of pre-operative antibiotic prophylaxis usage in elective surgical procedures in two teaching hospitals, Islamabad, Pakistan: An observational cross-sectional study

**DOI:** 10.1371/journal.pone.0231188

**Published:** 2020-04-07

**Authors:** Zakir Khan, Naveed Ahmed, Asim.ur. Rehman, Faiz Ullah Khan, Muhammad Saqlain, Maria Auxiliadora Parreiras Martins, Hazir Rahman

**Affiliations:** 1 Department of Pharmacy, Quaid-i-azam University Islamabad, Pakistan; 2 Department of Pharmacy, Administration and Clinical Pharmacy, School of Pharmacy, Health Science Centre, Xi'an Jiaotong University, Xi'an, China; 3 Faculdade de Farmácia, Universidade Federal de Minas Gerais, Belo Horizonte, Brazil; 4 Department of Microbiology, Faculty of Chemical and Life Sciences, Abdul Wali Khan University, Mardan, Pakistan; Northwestern University Feinberg School of Medicine, UNITED STATES

## Abstract

An audit of the antibiotic prophylaxis in surgical procedures is the basic area of antimicrobial stewardship programme. The current research aimed to evaluate the adherence-proportion of the pre-operative antibiotic prophylaxis (PAP) practices in common elective surgical procedures. It was an eight-month (January 2017 to August 2017) observational cross-sectional patients’ treatment record-based study conducted at two tertiary care teaching hospitals of Islamabad, Pakistan. We investigated the three most commonly performed elective general surgical procedures at the hospitals in adults aged > 18 years with no previous infection or surgery. The required data were extracted from the medical charts. Current prescribing practices were compared with the standard prescribing guidelines. A total of 660 (Government Hospital (GH), n = 330 and Private Hospital (PH), n = 330) procedures were observed. The most commonly performed elective general surgical procedures were laparoscopic cholecystectomy 307/660 (46.5%), followed by direct inguinal hernia 197/660 (29.8%) and total thyroidectomy 156/660 (23.6%). Non-use of PAP was observed in 64/660 (9.7%) cases. PAP was given to 90.3% (n = 596/660) cases (300/330 (90.9%) patients in GH and 296/330 (89.7%) in PH; P = 0.599). Based on the existing guidelines, the choice of antibiotics was correct in only 4.2% (25/596) patients (10/300; 3.3% cases at GH and 15/296; 5% at PH). The appropriate use of antibiotics was significantly greater in direct inguinal hernia (n = 19/193; 9.8%) cases compared with that in total thyroidectomy (n = 4/152; 2.6%) and laparoscopic cholecystectomy (n = 2/251; 0.8%) cases; P = 0.001. Compliance to the timing was only 51% (n = 304/596) of the total patients received PAP which was significantly lower in GH 97/300 (32.3%) as compared with that in PH 207/296 (69.9%); P = 0.001. Administration timing of antibiotics was observed to be more appropriate in total thyroidectomy (n = 79/152; 51.9%) cases than in laparoscopic cholecystectomy (n = 130/251; 51.8%) and direct inguinal hernia (n = 95/193; 49.2%) cases; P = 0.001. The route and dose were appropriate in accordance with the guidelines in all cases (100%). Most of the patients received ceftriaxone, a third-generation cephalosporin that is no longer recommended by the latest international guidelines. The current analysis revealed an alarmingly poor adherence rate with the guidelines in the three elective surgical procedures at both hospitals. To improve the situation, training and awareness programs about the antimicrobial stewardship interventions on the institutional level may be valuable.

## 1. Introduction

Surgical site infections (SSIs) are the most common complications in surgical procedures. SSIs account for increased morbidity, extended hospital stay and higher costs [1, 2]. The rate of SSIs varies significantly between health care settings and surgical categories [3]. According to previously conducted surveys, the United Kingdom (UK) and the United State of America (USA) have a prevalence rate of 16% and 31% respectively [4, 5]. A study conducted in Pakistan revealed that 13% of the patients who underwent elective surgery had encountered with SSIs [6]. Another recent study reported that the rate of SSIs was 6.5% in patients who underwent elective surgeries [7].

Pre-operative antibiotic prophylaxis (PAP) in surgical cases has been considered as an effective intervention to reduce the incidence of SSIs [1, 8, 9]. PAP should be appropriately administered in terms of selection, timing, dose, and route [10, 11]. An inappropriate choice of PAP can lead to higher cost and promotes resistance [10]. Bacterial resistance is an emerging public health threat and is responsible for dependence on more toxic, expensive, second-line therapeutic agents [2]. Moreover, the incorrect timing of PAP administration can cause a reduction in its efficacy [1, 10, 12].

Guidelines based on currently available clinical evidences are useful for the rational use of antibiotics to prevent the SSIs [10, 13–16]. Investigation of antibiotic prophylaxis is a crucial part of stewardship interventions [16]. Inappropriate use of antimicrobials was reported in the previously published literature and it is also one of the costliest categories of drug expenditure in the hospitals [17–19]. Furthermore, PAP comprises one-third of all the antibiotics used in the hospitals and 80% of all the antibiotics used in surgical procedures [1, 9, 20].

Inappropriate use of PAP, a high prevalence of multi-drug resistance, unavailability of local standard guidelines and limited available research on the utilization of PAP in Pakistani hospitals are still the main problems [6, 7, 21–23]. Based on the aforementioned explanation, we constructed a research hypothesis that limited PAP statistics, inappropriate prescription of PAP and unavailability of guidelines would lead to poor adherence with the standard treatment guidelines. Considering the importance of antibiotic prophylaxis, the present study aimed to evaluate the compliance of current prescribing practices with the standard treatment guidelines in selected elective surgeries.

## 2. Material and methods

### 2.1. Study design and setting

The current research was an observational cross-sectional patients’ treatment record-based study conducted within an eight-month (January 1^st^, 2017 to August 30^th^, 2017) timeframe. The study was carried out in the general surgery departments of Pakistan Institute of Medical Sciences (PIMS), a government-funded hospital (GH) and Shifa International Hospital (SIH), a private-funded hospital (PH) in Islamabad, Pakistan. GH is a 600-beds tertiary level teaching hospital serving a large population and has twenty-two medical and surgical centres. PH is also a tertiary care, multi-specialty 500-beds teaching hospital. Both hospitals provide medical facilities to the Rawalpindi and the Islamabad regions. These are also national-level referral hospitals for Northern areas of Azad Jammu and Kashmir, Khyber Pakhtunkhwa and Punjab, Pakistan.

### 2.2 Sample size, sampling technique and patient characteristics

The sample size was estimated in accordance with the recommendations of the World Health Organization/ International Network for Rational Use of Drugs (WHO/INRUD) methodology. According to the WHO/INRUD methodology, a sample size of at least 600 encounters/prescriptions of the patients is required to conduct a cross-sectional prospective study about current treatment practices [24]. Patients who underwent the three most common elective surgical procedures (laparoscopic cholecystectomy, direct inguinal hernia (DIH) and total thyroidectomy) were asked for consent to participate in the study. All cases of the selected elective surgical procedures were assessed during the study period. These were the common elective surgical procedures at the hospitals and performed in adult’s population. Laparoscopic cholecystectomy belongs to clean-contaminated wound surgery whereas; DIH and total thyroidectomy are the clean surgical interventions. Patients with emergency operations, any other elective surgical procedures, diagnosed with infectious disease, previous infection and surgical intervention, referred to other centres or those who had undergone surgeries in combination with another procedure were excluded from the study. A total of 705 patients consented to participate in the study. Forty-five (45/705; 6.4%) patients were excluded either due to incomplete medical records (20 patients) or having age less than 18 years (25 patients). Finally, 660 cases were included in the current study as per the inclusion criteria.

### 2.3. Data collection method and analysis

Important information was collected through a pre-designed detailed data collection proforma **(S1 File)**. Required information was gathered from the medical records, such as; name, medical record number, gender, age, weight, type of surgery, surgical intervention (duration of surgical procedure, date, duration of hospital stay) and antibiotic prophylaxis (class of antimicrobials/antibiotics, dose, timing of administration and the route of administration).

Data were evaluated according to the updated “Clinical Practice Guidelines for Antibiotic Prophylaxis in Surgery” [10] and Centers for Disease Control and Prevention Guideline” for the prevention of SSIs [13]. Surgical teams at teaching hospitals of Pakistan are expected to adhere to the American-based recommendations for the best possible practice due to absence of up-to-date consensed national or hospital-based guidelines for PAP.

### 2.4. Recommended PAP treatment course

The above-mentioned guidelines emphasized on the use of appropriate and inexpensive narrow-spectrum PAP as an intravenous single dose and also to be administered within 30 to 60 minutes before the first surgical incision. According to the guidelines, cefazolin is the first-line drug of choice for the laparoscopic cholecystectomy and DIH however, if the patient is allergic to the beta-lactams then vancomycin, clindamycin or gentamicin should be the appropriate alternative drug for elective surgical procedures. The guidelines advocate the non-usage of PAP in total thyroidectomy cases [10, 13]. The detailed recommended PAP treatment course for these three types of surgeries is presented in **S1 Table**.

### 2.5. Appropriateness of preoperative antibiotic prophylaxis practices

Appropriateness of PAP practices was evaluated according to the following criteria based on the aforementioned guidelines [10, 13]: (a) Was the choice of PAP appropriate and in accordance with the surgical procedure? (b) Was an appropriate agent used as PAP? (c) Was the administration timing of PAP appropriate? (d) Was the dose of PAP correct? and (e) Was PAP administered through a proper route?

If any of the assessed criteria not fulfilled then PAP prescriptions were measured inappropriate and were considered as non-adherence with the guidelines. Anatomical Therapeutic Chemical (ATC) classification system was employed to document the utilization pattern of the most common classes and combinations of antibiotics used in this study [25].

### 2.6. Statistical analysis

Collected data were encoded in Microsoft Excel version 2016 and SPSS 20.0. Descriptive statistics were used as frequencies and percentages for categorical variables and mean and standard deviation for numerical parameters. To find the association between antimicrobial use and sample characteristics, Chi-square test for categorical variables and Kruskal-Wallis test for continuous variables were used. Results were expressed as numbers and proportions and p-value of less than 0.05 (p<0.05) was considered significant.

### 2.7. Ethical approval

The study was performed in accordance with the declaration of Helsinki. The Bio-Ethical committee of Quaid-i-Azam University, Islamabad, Pakistan (No. DFBS/2016-623; dated: December 22, 2016) and the ethical/institutional review boards of GH (No. F.1-1/2015/ERB/SZABMU/; dated: August 18, 2016) and PH (No. IRB-637-085-2016; dated: August 15, 2016) approved the study protocols. All methods were performed in accordance with the relevant guidelines and regulations. Patients were invited to participate in the study and they signed informed consent at recruitment.

## 3. Results

A total of 660 surgical procedure cases were observed, including 330 (laparoscopic cholecystectomy n = 148, DIH n = 113 and total thyroidectomy n = 69) cases from GH and 330 (laparoscopic cholecystectomy n = 159, DIH n = 84 and total thyroidectomy n = 87) from PH. The mean age of the patients was 43.2 years. Male patients were more than female (54.8% versus 45.2%). Details of the demographics are given in **Table 1.**

**Table 1:** Patients demographics (n = 660).

Hospitals	GH			Total	P-Values	PH			Total	P-Values
Surgeries	Laparoscopic cholecystectomy n (%)	DIH n (%)	Total thyroidectomy n (%)			Laparoscopic cholecystectomy n (%)	DIH n (%)	Total thyroidectomy n (%)		
**Gender** *					**0.351**					**0.17**
**Males**	76 (51%)	82 (73%)	17 (25%)	**175 (53%)**		83 (52%)	66 (79%)	38 (44%)	187 (57%)	
**Females**	72 (49%)	31 (27%)	52 (75%)	**155 (47%)**		76 (48%)	18 (21%)	49 (56%)	143 (43%)	
Age (Years)*					**0.086**					**0.128**
**18–30**	39 (26.4%)	49 (43.4%)	35 (51%)	123 (37.2%)		23 (14%)	14 (16.7%)	28 (32.2%)	65 (19.7%)	
**31–40**	44 (30%)	31 (27.4%)	19 (27.5%)	94 (28.5%)		26 (16%)	20 (23.8%)	14 (16%)	60 (18%)	
**41–50**	21 (14%)	14 (12.4%)	7 (10%)	42 (12.7%)		24 (15%)	20 (23.8%)	11 (12.6%)	55 (16.6%)	
**51–60**	29 (19.5%)	10 (8.8%)	3 (4.4%)	42 (12.7%)		40 (25%)	15 (17.9%)	13 (15%)	68 (26.6%)	
**61–70**	9 (6%)	5 (4.4%)	4 (5.8%)	18 (5.4%)		27 (17%)	14 (16.6%)	14 (16%)	55 (16.6%)	
**‹ 70**	6 (4%)	4 (3.5%)	1 (1.4%)	11 (3.3%)		19 (12%)	1 (1%)	7 (8%)	27 (8.1%)	
**Total**	148 (100%)	113 (100%)	69 (100%)	330 (100%)		159 (100%)	84 (100%)	87 (100%)	330 (100%)	
Weight (Kg)**					**0.418**					**0.634**
Mean (SD)	74.9 (10.8)	72.5 (10.0)	69.3 (8.9)			76.3 (9.6)	75.5 (9.2)	73.1 (11.7)	-	-
Range	53–107	54–105	55–95			54–104	45–97	47–103		
**Duration of stay (days)** **					**0.235**					**0.196**
Mean (SD)	3.46 (.52)	2.81 (.51)	2.38 (.48)			3.04 (.57)	2.39 (.49)	2.17 (3.8)		
Range	2–4	2–4	2–3		-	2–4	2–3	2–3	-	-

*Chi-Square tests

**Kruskal-Wallis tests, GH, government hospital; PH, private hospital; DIH, direct inguinal hernia; n, number; SD, standard deviation; h, hours; Kg, Kilogram.

### 3.1. Evaluation of PAP Practices and association between PAP and sample characteristics

#### 3.1.1. PAP indication

PAP was given in 90.3% (n = 596) of the surgical procedures and about 9.7% (n = 64) patients did not receive any PAP **(Tables 2 and 3)**. PAP was given to 300 (90.9%) patients in GH and 296 (89.7%) in PH **(Table 2)**. There was no statistically significant difference found between the use of PAP and hospital setting (P = 0.599) **(Table 3).**

**Table 2:** Pre-operative antibiotic prophylaxis (PAP) practices among selected surgeries (n = 660).

PAP Practices	Laparoscopic cholecystectomy n (%)	GH DIH n (%)	Total thyroidectomy n (%)	Total	Laparoscopic cholecystectomy n (%)	PH DIH n (%)	Total thyroidectomy n (%)	Total
**Surgical procedures**	148 (44.8)	113 (34.2)	69 (20.9)	330 (100)	159 (48.2)	84 (25.4)	87 (26.3)	330 (100)
**Antimicrobial given**	123/148 (83.1%)	109/113 (96.4%)	68/69 (98.5%)	300/330 (90.9%)	128/159 (80.5%)	84/84 (100%)	84/87 (96.5%)	296/330 (89.7%)
**Antimicrobial correct choice**	2/123 (1.6%)	7/109 (6.4%)	1/68 (1.4%)	10/300 (3.3%)	0/128 (0%)	12/84 (14.3%)	3/84 (3.5%)	15/296 (5%)
**Route**								
Intravenous (IV)	123 (100%)	109 (100%)	68 (100%)	300 (100%)	128 (100%)	84 (100%)	84 (100%)	296 (100%)
**Timing**								
30–60 minutes before SI	39/123 (31.7%)	36/109 (33%)	22/68 (32.3%)	97/300 (32.3%)	91/128 (71.1%)	59/84 (70.2%)	57/84 (67.8%)	207/296 (69.9%)
More than 30–60 min	84/123 (68.3%)	73/109 (67%)	46/68 (67.6%)	203/300 (67.7%)	37/128 (28.9%)	25/84 (29.8%)	27/84 (32.1%)	89/296 (30.1%)

PAP, Pre-operative antibiotic prophylaxis; GH, government hospital; PH, private hospital; DIH, direct inguinal hernia; SI, Surgical incision; n, number; IV, Intravenous.

**Table 3:** Comparison of association of antimicrobial use and sample characteristics between two hospitals (n = 660).

Variables	GH (n = 330)			PH (n = 330)			Total sample (n = 660)		
Variables	Antimicrobial use		P-Value^a^	Antimicrobial use		P-value	Antimicrobial use		P-value ^a^
	Yes	No		Yes	No		Yes	No	
Gender*			**0.464**			**0.409**			**0.278**
Male	161 (92.0%)	14 (8.0%)		170 (90.9%)	17 (9.1%)		331 (91.4%)	31 (8.6%)	
Female	139 (98.1%)	16 (10.3%)		126 (88.1%)	17 (11.9%)		265 (88.9%)	33 (11.1%)	
Type of surgery*			**<0.001**			**<0.001**			**<0.001**
Laparoscopic cholecystectomy	123 (83.1%)	25 (16.9%)		128 (80.5%)	31 (19.5%)		251 (81.8%)	56 (18.2%)	
DIH	109 (95.5%)	4 (3.5%)		84 (100%)	0 (0.0%)		193 (98.0%)	4 (2.0%)	
Total thyroidectomy	68 (95.6%)	1 (1.4%)		84 (96.6%)	3 (3.4%)		152 (97.4%)	4 (2.6%)	
**Hospital type**			**0.599**			**0.599**			**0.599**
**Total**	300 (90.9%)	30 (9.1%)		296 (89.7%)	34 (10.3%)		596 (90.3%)	64 (9.7%)	

GH, government hospital; PH, private hospital; DIH, direct inguinal hernia^.^

^a^ P<0.05 (2-tailed) considered significant using Chi square test.

#### 3.1.2. PAP choice

Based on the existing guidelines, the choice of antibiotics was correct in only 4.2% (25/596) patients (10/300; 3.3% at GH and 15/296; 5% at PH) **(Table 2).** The most commonly prescribed antimicrobial was ceftriaxone (n = 342; 51.8%) followed by amoxicillin plus clavulanic acid (n = 98; 14.8%) and cefuroxime (n = 62; 9.4%). However, cefazolin was administered to only 5.1% (n = 34) and vancomycin to 0.45% (n = 3) cases as PAP **(Table 4)**. Use of appropriate choice of PAP was greater in DIH (n = 19/193; 9.8%) than total thyroidectomy (n = 4/152; 2.6%) and laparoscopic cholecystectomy (n = 2/251; 0.8%) cases **(Table 2).**

**Table 4:** Frequency and percentages of various PAP prescribed in three commonly performed elective surgeries (n = 660).

Pre-operative antimicrobials (dose)	WHO/ATC code	GH			PH	
		n	%		n	%
**Laparoscopic cholecystectomy**						
Ceftriaxone 2g	J01DD04	103		69.6	104	65.4
**Non-use of PAP**	-	25		16.9	31	19.5
Cefoperazone+sulbactam 1g	J01DD62	18		12.1	-	-
*Cefazolin 2g	J01DB04	2		1.2	-	-
Cefuroxime 1.5g	J01DC02	-		-	7	4.4
Amoxicillin+clavulanic acid 1.2 g	J01CR02	-		-	6	3.8
Ciprofloxacin 500 mg	J01MA02	-		-	6	3.8
Piperacillin+sulbactam 4.5g	J01CR05	-		-	4	2.5
Vancomycin 500mg	J01XA01	-		-	1	0.6
**Total**	**-**	148		100	159	100
**DIH**						
Ceftriaxone 2g	J01DD04	64		56.6	43	51.2
Cefuroxime 1.5g	J01DC02	20		17.7	15	17.9
Azithromycin 500 mg	J01FA10	13		11.5	5	6
*Cefazolin 2g	J01DB04	7		6.2	10	11.9
Amoxicillin+clavulanic acid 1.2 g	J01CR02	3		2.7	8	9.5
**Non-use of PAP**	-	4		3.5	-	-
Amikacin 500 mg	J01GB06	2		1.8	-	-
**Vancomycin 500mg	J01XA01	-		-	2	2.4
Piperacillin+sulbactam 4.5g	J01CR05	-		-	1	1.2
**Total**	-	113		100%	84	100
**Total thyroidectomy**						
Amoxicillin+clavulanic acid 1.2 g	J01CR02	32		46.4%	49	56.3%
Cefuroxime 1.5g	J01DC02	14		20.3%	6	6.9%
Ceftriaxone 2g	J01DD04	10		14.5%	18	20.7%
Amikacin 500 mg	J01GB06	6		8.7%	-	-
Cefazolin 2g	J01DB04	4		5.8%	11	12.6%
Cefradine 500mg	J01DB09	2		2.9%	-	-
*Non-use of PAP	-	1		1.4%	3	3.4%
**Total**	-	69		100%	87	100%

* 1^st^ choice of drug

** 2^nd^ choice of drug, GH, government hospital; PH, private hospital; DIH, direct inguinal hernia; n, number; WHO/ATC, World Health Organization/anatomical therapeutic classification.

#### 3.1.3. Dose of PAP

Regarding the dose, a 2-gram of ceftriaxone and cefazolin, 1.5g of cefuroxime and 1.2 g of amoxicillin plus clavulanic acid were administered as PAP. Details about the dose of PAP are shown in **Table 4**.

#### 3.1.4. PAP Route and timing of administration

All patients received PAP through the intravenous route and only 51% (n = 304/596) of the total patients received PAP according to the recommended timing of the administration **(Table 5).** Compliance concerning the timing was significantly lower in GH 97/300 (32.3%) as compared to PH 207/296 (69.9%); P = 0.001 **(Table 2).** Timing of administration of antibiotics was observed more appropriate in total thyroidectomy (n = 79/152; 51.9%) than laparoscopic cholecystectomy (n = 130/251; 51.8%) and direct inguinal hernia (n = 95/193; 49.2%) surgery; P = 0.001 **(Table 2).** Further details about the use of PAP Practices with respect to the surgical procedure are also summarized in Tables 2, 3, 4 and 5.

**Table 5:** Administration time of PAP according to the procedures (n = 660).

Time of administration	GH hospital n (%)	PH hospital n (%)
**Laparoscopic cholecystectomy**		
30–60 minutes before SI	39 (31.7%)	91 (71.1%)
61–120 minutes before SI	65 (52.8%)	26 (20.3%)
121–180 minutes before SI	16 (13%)	8 (6.2%)
181–240 minutes before SI	3 (2.4%)	3 (2.3%)
**Total PAP use**	123 (83.1%)	128 (80.5%)
No PAP given	25 (16.9%)	31 (19.5%)
***Total Cases***	***148 (100%)***	***159 (100%)***
**DIH**		
30–60 minutes before SI	36 (33%)	59 (70.2%)
61–120 minutes before SI	48 (44%)	18 (21.4%)
121–180 minutes before SI	18 (16.5%)	5 (5.95%)
181–240 minutes before SI	6 (5.5%)	1 (1.2%)
241–300 minutes before SI	1 (0.9%)	1 (1.2%)
**Total PAP use**	109 (96.5%)	84 (100%)
No PAP given	4 (3.5%)	0 (0)
***Total Cases***	***113 (100%)***	***84 (100%)***
**Total Thyroidectomy**		
30–60 minutes before SI	22 (32.3%)	57 (67.8%)
61–120 minutes before SI	39 (57.3%)	22 (26.2%)
121–180 minutes before SI	4 (5.9%)	4 (4.7%)
181–240 minutes before SI	3 (4.4%)	0 (0%)
241–300 minutes before SI	0 (0%)	1 (1.2%)
**Total PAP use**	68 (98.5%)	84 (96.5%)
No PAP given	1 (1.5%)	3 (3.5%)
***Total Cases***	***69 (100%)***	***87 (100%)***

SI, surgical incision; GH, government hospital; PH, private hospital; DIH, direct inguinal hernia; n, number.

## 4. Discussion

Adherence of the prescribing practices with the recommendations of evidence-based clinical practice guidelines was analysed in the present study. Appropriate use of PAP is a proven strategy in decreasing surgical infections. Overall, in this study, PAP was administered in 90.3% cases. However, the appropriate antibiotic prophylaxis was used in only 4.2% of the cases. These findings revealed an alarming poor compliance rate in contrast to the findings of different global studies. The compliance rate in other countries such as USA (99%)[26], Israel (97%) [27], Greece (70%)[28], Philippines (44%) [2] and Palestine (18.5%)[14] is much better than that in Pakistan.

There are two possible reasons for the lower compliance rate; first is not giving the PAP when it is recommended like in 12% (60 out of 504) cases of laparoscopic cholecystectomy and DIH in which the guidelines advocate the use of PAP but were not given; the second is the use of PAP when it is not recommended by the guidelines as in 97% (152 out of 156) cases of total thyroidectomy. Both conditions have harmful consequences in the patients. In the first situation, non-use of the recommended antibiotics results in an increased vulnerability to wound development, which might lead to an increased hospital stay, morbidity and mortality rate [1, 29]. In the second scenario, the use of PAP when it is not recommended can lead to serious side effects, e.g. *Clostridium difficile* infection [30, 31]. The possible explanation of low adherence with the guidelines might be due to the absence of updated national guidelines and that the surgeons were following non-standard or non-official routine protocols instead of international standard prescribing guidelines [32].

In this study, the most commonly prescribed antibiotic for prophylaxis was ceftriaxone. The antibiotics that are recommended by the guidelines in elective surgical procedures were rarely prescribed. Higher utilization of ceftriaxone as PAP was also reported in Ethiopia [1], Turkey [33], Malaysia [34] and Jordan [35]. The third generation cephalosporins and other broad-spectrum antibiotics are not required for prophylaxis. These agents have less activity against staphylococcal infections, high rates of resistance development, as well as an increased financial burden on the patients as compared to cefazolin [36]. Moreover, the emergence of extended-spectrum beta-lactamase (ESBL) producing microorganisms is due to the overuse of third-generation cephalosporins [37]. It is evident that, to avoid SSIs in elective surgeries, cefazolin is enough to cover the pathogens, hence there is no need for multiple antibiotic therapies [10]. These results indicate that some surgeons are not keeping up-to-date standards of best practices [1].

Inappropriate timing of PAP was another problem observed in the current study and results showed that only 51% of the patients received PAP at the recommended time. These findings were comparable to the studies conducted in Ethiopia [1] and the Philippines [2]. Higher adherence rate with PAP timing was reported in studies carried out in Australia (93%) [38] and Palestine (60%) [14]. A study conducted in Israel reported 32% [28] adherence rate, which was lower as compared with the present study. Less protection against microbial flora was given to the patients who did not receive antibiotics at the optimal time, as described in the literature [10, 12, 13]. The inappropriate use of PAP in the present study can be due to several reasons. These include surgeons’ thinking or fear of acquiring infection after surgery, unavailability of a clinical pharmacist, un-trained resident-in-charge and lack of medication protocols and treatment guidelines. A clinical pharmacist can play a vital role in assisting the surgeons to choose the correct medications according to the guidelines. Proper training of the resident in-charge on the prescribing practices can also enhance the rational use of medicines. Noncompliance with the guidelines in the current study raised an important issue, which needs to be addressed. There is a difference in prescribing practices according to the hospital's complexity. Greater discrepancies could be expected at primary and secondary care units because most of them do not have a pharmacy and therapeutic committees and infection control facilities.

The prescribing practice is a complex phenomenon. The current study had access to well-reported data on PAP practices and was adequately powered. Some limitations must be acknowledged. First, the present study only focused on the compliance rate of PAP in the three commonly performed elective surgical procedures. Compliance with the guidelines may be affected by many factors, such as cultural, educational, training, prescribers-pharmacist and nurse influences and drug supply and administrative problems. These aspects were not evaluated, and such studies could provide important contributions to support the interventions. Second, the post-surgical infection rate was not determined in the current study. Therefore, the authors do not know if the nonadherence to the guidelines had any clinical consequences. Fourth, this study uses published recommendations of international clinical practice guidelines for antibiotic prophylaxis in surgery to measure appropriateness according to evidence-based international standards. There was no local consensed guideline available in both hospitals. Clinical guidelines emphasized on and present the best evidence available to the experts. However, following the guideline recommendations will not necessarily result in the best outcome. Guidelines can never replace clinical expertise when treatment decisions for individual patients are being taken. Guidelines help to focus on decisions. Clinical decisions must also take into account, the patients’ values and preferences and their circumstances. Therefore, due to different personal values, preferences and individual circumstances of the present patients and country, a possibility exists that the recommendations given by the guidelines were not practicable in these patients or for the situation in Pakistan. Finally, these findings do however add a piece of useful information, particularly around appropriate PAP use, adherence with the standard guidelines and health systems in developing countries.

## 5. Conclusion

In the present study, it was concluded that adherence with the detailed recommendations related to the antibiotics’ choice and timing was alarmingly poor. Prescribing practices in elective surgeries require improvement in various areas. Compliance to the guidelines by the surgeons remained a challenge, as reported in previous studies around the globe and also in the present study. The findings also emphasized that prescribing practices should be periodically evaluated. Urgent implementation of the antimicrobial stewardship program, continued medical education and placement of guidelines in the operating rooms can be the important interventions. Checking of prescription, dose, timing of antibiotics and other medicines by a clinical pharmacist at the time of order can be a more active intervention. Furthermore, studies needed to be carried out to access the effectiveness of such interventions and the effect of non-compliance on the development of SSIs.

## Supporting information

S1 File Data collection proforma used for the study. (DOC)

S1 Table PAP treatment course for selected surgical procedures. (DOC)
