# Supplementary material for: Audit of pre-operative antibiotic prophylaxis usage in elective surgical procedures in two teaching hospitals, Islamabad, Pakistan: An observational cross-sectional study
Source: PLoS One. 2020 Apr 7;15(4):e0231188. doi: 10.1371/journal.pone.0231188 (PMC7138312; doi:10.1371/journal.pone.0231188)
Supplement: S1 File — (DOC) [file pone.0231188.s001.doc]

| 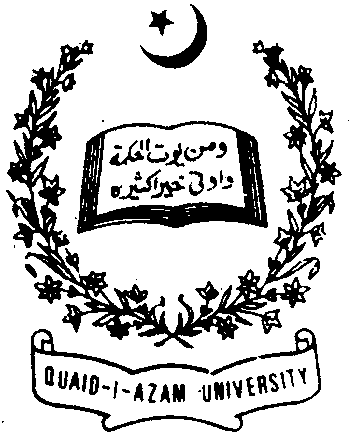 | Department of Pharmacy  QUAID-I-AZAM UNIVERSITY,  ISLAMABAD, PAKISTAN  Tel: +92-051-90644056 |
| --- | --- |

## Data Collection Proforma

This data extraction format is prepared to retrieve data from patient charts on the utilization pattern of antibiotic prophylaxis for surgical site infections at Pakistan institute of Medical Sciences and Shifa international hospital, Islamabad from January,1, 2017 to August 30, 2017.

## 1. Medical and surgical history of patients underwent surgery

| 1.1 | Type of surgery | 1. clean  2. clean contaminated |
| --- | --- | --- |
| 1.2 | Most common surgical procedure performed for the patient | 1.Laparoscopic cholecystectomy  2. Direct inguinal hernia  3. Total thyroidectomy |
| 1.3 | Co-morbid condition does the patient has? | 1.DM  2. HIV-AIDS  3.TB  4.Hypertetion  5.Malignancy  6.Other comorbidity________ |
| 1.4 | Previous infection and surgery | 1. Yes 2. No |

## 2. Surgical antibiotics usage

| 2.1 | Type of wound that the patient | 1.Clean  2.Clean contaminated  3.Contaminated  4.Dirty |
| --- | --- | --- |
| 2.2 | Antibiotic prescribed or not? | 1.Yes  2.No |
| 2.3 | If Yes, type of antibiotic given to the patient as prophylaxis | 1. Ceftriaxone 2. Cefazolin 3. Cefuroxime 4. Metronidazole 5. Others------ |
| 2.4 | The form of antibiotics | 1.single  2.combined |
| 2.5 | Duration of treatment (in hours) | ----------- |
| 2.6 | Dose of antibiotics | ------------- |
| 2.7 | Dosage form of antibiotics | --------------- |
| 2.8 | Route of administration | 1.intravenous  2.intramuscular  3. intradermal  4.other |
| 2.9 | Timing of administration | 1.within 30-60 minutes before surgical incision  2. More than 1 hour before surgical incision |

1. **Adherence with standard treatment guidelines**

| 3.1 | Is choice of SAP appropriate according to guidelines? | 1.Yes  2. No |
| --- | --- | --- |
| 3.2 | Appropriate dose? | 1.Yes  2. No |
| 3.3 | Route of administration? | 1.Yes  2. No |
| 3.4 | Duration of SAP? | 1.Yes  2. No |
| 3.5 | Is timing of administration before surgical incision appropriate? | 1.Yes  2. No |
