# Supplementary material for: Audit of pre-operative antibiotic prophylaxis usage in elective surgical procedures in two teaching hospitals, Islamabad, Pakistan: An observational cross-sectional study
Source: PLoS One. 2020 Apr 7;15(4):e0231188. doi: 10.1371/journal.pone.0231188 (PMC7138312; doi:10.1371/journal.pone.0231188)
Supplement: S1 Table — (DOC) [file pone.0231188.s002.doc]

**Supplementary File 1.** Summary of Guidelines recommendations for surgeries.

| Procedures | Type of wound | Antibiotic  Choice | Antibiotic use and route | Dose | Timing (min) |
| --- | --- | --- | --- | --- | --- |
| Laparoscopic cholecystectomy | Clean contaminated |  |  |  |  |
|  |  | First line | Cefazolin IV | 2g | 60 |
|  |  | Alternative | Clindamycin IV | 900 mg |  |
|  |  |  | Gentamicin IV | 5mg/kg | 60 |
| DIH | Clean |  |  |  | 60 |
|  |  | First line | Cefazolin IV | 2g | 60 |
|  |  | Alternative | Clindamycin IV | 900 mg | 60 |
|  |  |  | Vancomycin | 15 mg/kg | 120 |
| Total thyroidectomy | Clean |  | No need of Antibiotic prophylaxis |  |  |

*Legends: DIH* Direct Inguinal Hernia*, Min* Time*, WHO* World health Organization, *IV* Intravenous, *Mg* Milligram, *Kg* Kilogram
